# ARNI Pre-Operative Use and Vasoplegic Syndrome in Patients Undergoing Heart Transplantation or Left Ventricular Assist Device Surgery

**DOI:** 10.3390/medsci10010002

**Published:** 2021-12-21

**Authors:** Lamis Haider, Elisabeth Hugon-Vallet, Jean Philippe Constantin, Zakaria Riad, Laurent Sebbag, Nathan Mewton

**Affiliations:** 1 Heart Failure Department, Hospices Civils de Lyon, Claude Bernard University Lyon 1, Hôpital Cardio-Vasculaire Louis Pradel, 69003 Lyon, France; elisabeth.hugon-vallet@chu-lyon.fr (E.H.-V.); constantin.jp@gmail.com (J.P.C.); laurent.sebbag@chu-lyon.fr (L.S.); 2 Cardiac Critical Care, Hospices Civils de Lyon, Claude Bernard University Lyon 1, Hôpital Cardio-Vasculaire Louis Pradel, 69003 Lyon, France; zakaria.riad@chu-lyon.fr; 3 Centre d’Investigation Clinique Inserm 1407, Heart Failure Department, Carmen Inserm 1060, Hospices Civils de Lyon, Claude Bernard University Lyon 1, Hôpital Cardio-Vasculaire Louis Pradel, 69003 Lyon, France

**Keywords:** vasoplegic syndrome, angiotensin receptor neprilysin inhibitors, heart transplantation, left ventricular assist-device therapy

## Abstract

**Background**: Vasoplegic syndrome after orthotopic heart transplantation (OHT) or left ventricular assist device (LVAD) implantation is a rare but highly lethal syndrome with complex etiologies. The objective of this study was to assess if the preoperative use of sacubitril-valsartan combination is associated with an increased vasoplegic syndrome (VS) frequency after OHT or LVAD implantation and its relationship with 30-day mortality. **Methods:** A retrospective review of perioperative data, between January 2016 and December 2017, from 73 consecutive OHT and LVAD surgery adult patients at our institution was performed. VS was defined as normal cardiac output with persistent low systemic resistance requiring a norepinephrine intravenous perfusion > 0.5 µg/kg/min and the absence of sepsis or hemorrhagic shock within 48 h after surgery. Patients were all followed-up for adverse events and all-cause mortality at 30 days. **Results:** In our cohort of 73 patients (median age 51.7 years, 65% male patients), 25 (34%) patients developed VS. Twenty-two (30.1%) patients were on ARNI at the time of surgery, 31 (42.5%) were on other RAS blockers, 12 (16.4%) were on norepinephrine and 8 (11%) had no pre-operative drug. The pre-operative use of any vasoactive agent, was not significantly associated with VS (OR = 1.36; IC95% [0.78; 2.35]; *p =* 0.38). The pre-operative use of an ARNI compared to all other groups was not significantly associated with VS (OR = 2.0; IC95% [0.71; 5.62]; *p* = 0.19). The pre-operative use of an ARNI compared to other RAS blockers was also not significantly associated with VS (OR = 1.25; IC95% [0.37; 4.26]; *p* = 0.72). At 30 days, 18 (24.7%) patients had died. The pre-operative treatment with ARNI, or other RAS inhibitors was associated with a significantly lower rate of death compared to the absence of treatment (HR = 0.11; IC95% [0.02; 0.55]; *p* = 0.009 for ARNI and HR = 0.20; IC95% [0.06; 0.69]; *p* = 0.011 for other RASi). **Conclusions:** Preoperative use of sacubitril-valsartan was not significantly associated with development of vasoplegic syndrome in patients undergoing OHT or LVAD surgery. Furthermore, our data suggests a significant 30-day survival benefit with efficient renin-angiotensin blockade before surgery.

## 1. Introduction

The vasoplegic syndrome (VS) after cardiac surgery includes refractory hypotension reduced systemic vascular resistance and normal or elevated cardiac output. The VS occurs with an incidence of 8–25% in the general cardiac surgery population. It is significantly more prevalent following heart transplantation (HTx) and occurs in up to 54% of cases [1,2]. Reports have suggested that the VS was associated with the increased pro-inflammatory state in advanced heart failure patients, combined with the inflammatory response following reperfusion and the release of donor antigens [3].

The VS is associated with poor clinical outcomes and worse overall prognosis. It is associated with prolonged intensive care and hospital stay and higher mortality [4,5].

VS pathophysiology is not completely understood. It involves the activation of several vasodilator pathways in addition to a decreased response to exogenous and endogenous vasopressors [6]. Some reports have shown the association of pre-operative vasodilators with increased VS incidence [7].

In 2016, ARNI (angiotensin receptor and neprilysin inhibitor) was incorporated into clinical practice guidelines after showing its superiority against enalapril in patients with heart failure and reduced ejection fraction. ARNIs are being increasingly used in patients with advanced heart failure and candidates to HTx [8,9]. Compared to ACE-inhibitors, ARNIs have a significantly increased vasodilatory and hypotensive effect. There is little data about its association with the incidence of VS in patients undergoing HTx or left ventricular assist device (LVAD) implantation [10].

This study assessed the association of pre-operative use of ARNIs with VS incidence compared to other renin-angiotensin system inhibitors in patients undergoing HT or LVAD implantation. As a secondary objective, we also assessed the association of pre-operative ARNI use with the 30-day mortality rate following HT or LVAD implantation.

## 2. Methods

### 2.1. Study Design and Population

This was a retrospective study on all adult patients (>18 years) who underwent orthotopic heart transplantation or a durable LVAD implantation at our institution, a tertiary referral university hospital. The study period extended from the 1 January 2016 to the 31 December 2017. 

The study protocol was approved by our institutional ethics committee and waived the requirement for obtaining a signed informed consent for all participants.

Patient demographics, perioperative course and outcome data including the incidence of complications, length of hospital and intensive care unit (ICU) stay, and survival data were retrospectively retrieved from our surgical, transplant, and intensive care clinical electronic medical file databases. 

### 2.2. Vasoplegic Syndrome Definition

The vasoplegic syndrome was defined as the association of the following characteristics within 48 h of heart transplantation or LVAD implantation:

- normal cardiac function by a transthoracic echocardiogram (left ventricular ejection fraction 55% or greater),
- cardiac index > 2.2 L/min/m^2^
- requirement a norepinephrine intravenous perfusion > 0.5 µg/kg/min for more than 24 h in the first 48 h postoperatively to maintain a mean arterial pressure ≥ 65 mm Hg
- and the absence of documented sepsis or hemorrhagic shock.

There is currently no standard consensus for the definition of the VS. Our definition has been consistently used at our institution in routine practice and is based on measurements directly obtained to accurately characterize the vasodilatory state. This definition has been reported in prior publications in this field of research [5,11,12,13].

Management of vasopressor agents was performed by the cardiac surgery intensive care unit staff. Patients with hypotension secondary to cardiogenic shock, sepsis, hypovolemia, or other etiologies were excluded. 

The preoperative treatment with vasoactive effects was collected and classified into four categories:

- ARNI
- Other Renin-angiotensin blockers (ACEi and ARBs)
- Norepinephrine
- Absence of any vasoactive pre-operative treatment (reference group)

### 2.3. Study Endpoints

The primary objective of this study was to assess the association between the prescription of angiotensin receptor neprilysin inhibitor (ARNI), angiotensin-converting enzyme (ACE) inhibitors, or angiotensin II receptor blockers (ARBs) preoperatively, and the occurrence of postoperative vasoplegic syndrome. 

The secondary objectives were to assess the association between each preoperative treatment and: 30-day postoperative mortality, duration of hospitalization in the intensive care unit, duration of invasive ventilation, need for renal replacement therapy during hospitalization, the occurrence of mesenteric ischemia or any ischemia, maximum dose of noradrenaline, duration of treatment with noradrenaline, use of secondary vasoconstrictor such as methylene blue and/or terlipressin. 

### 2.4. Statistical Analysis

All analyses for this study were exploratory. Categorical variables were compared between the VS+ and VS− patients using the chi-square or Fisher’s exact tests and expressed as a percentage. Continuous variables were compared using the Student T-test if parametric or the Wilcoxon rank-sum test if non-parametric and presented as means ± standard deviation or medians and interquartile range. When there were more than two groups for continuous data, a simple analysis of variance was performed and the corresponding *p*-value for the F-test presented. No further post-hoc intergroup comparisons were performed to limit the level of alpha risk inflation. 

The survival analyses were performed using time-to-event data (censored date was the last follow-up time at 30-days) and are displayed using the Kaplan-Meier method and compared between VS+ and VS− groups and the different pre-operative vasoactive treatment groups with the log-rank test. Univariate Cox regression models were used to assess the association between 30-day mortality and VS status, and also pre-operative vasoactive treatment. Pre-surgery norepinephrine use was separated from RASi use in these analyses. 

Univariable logistic regression analysis was performed to assess the relationship between pre-operative vasoactive treatment and the occurrence of VS. Then, this association was analyzed in a multivariate logistic regression model after adjustment for other important clinical confounders: age, type of surgery (LVAD/HT), surgical extra-corporeal circulation time, and LVEF < 35%. There was no correction for multiple testing. For all regression analyses, the patient group without any vasoactive drug prior to surgery was defined as the reference group. 

Statistical significance was defined as a *p*-value < 0.05 and all tests were two-sided. All statistical analyses were performed using the STATA software (version 14; StataCorp, College Station, TX, USA).

## 3. Results

### 3.1. Baseline Characteristics

A total of 73 patients with 54 HT and 19 durable LVAD surgeries were performed during the study period. The median age was 51.7 years, with 65% male patients. All baseline characteristics are presented in Table 1. Of those, 25 patients (34%) met the criteria for vasoplegic syndrome (VS+) and 48 patients (66%) did not (SV−). 

Twenty-two (30.1%) patients were on ARNI at the time of surgery, 31 (42.5%) were on other RAS blockers, 12 (16.4%) were on norepinephrine and 8 (11%) had no vasoactive pre-operative treatment.

VS+ and VS− patients baseline characteristics are also summarized in Table 1. There were no significant differences between VS+ and VS− patients at baseline. 

There were no significant differences in the perioperative variables between VS− and VS+ patients.

Pre-surgery therapy with ARNI, RAS, β-blockers, phosphodiesterase inhibitors, or calcium channel blockers, were not found to be statistically different between groups (*p* = 0.3). In VS+ patients, 10 (40%) were pretreated by ARNI, seven (28%) by ACE inhibitor or ARBs, five (20%) by norepinephrine and three (12%) had no vasoactive agents (*p* = 0.3). Baseline characteristics of each vasoactive category are reported in the supplemental data for this manuscript (Table S1 in Supplementary Materials).

### 3.2. Pre-Operative Treatment Use and VS

The pre-operative use of any vasoactive agent was not significantly associated with VS (OR = 1.36; IC95% [ 0.78; 2.35]; *p =* 0.38). The pre-operative use of an ARNI compared to all other groups was not significantly associated with VS (OR = 2.0; IC95% [0.71; 5.62]; *p* = 0.19). The pre-operative use of an ARNI compared to other RAS blockers was also not significantly associated with VS (OR = 1.25; IC95% [0.37; 4.26]; *p* = 0.72).

In the multivariate logistic regression analysis adjusting for age, LVEF, surgery type, extracorporeal circulation time, the pre-operative use of an ARNI was also not significantly associated with VS (OR = 2.18; IC95% [0.35; 13.59]; *p* = 0.40). 

Patients pretreated with ARNI have a longer postoperative norepinephrine treatment duration (7.1 days) than patients pretreated with ACEi/ARBs (4.7 days) or No RASI (3.9). However, this difference is not significant. (Table 2)

### 3.3. Vasoplegia and Adverse Outcomes

All-cause mortality at 30 days in our study population was 24.7% (*n* = 18). 

Vasopressin was used in 4(16%) VS+ patients and methylene blue in 2(8%) VS+ patients. There was no use of phosphodiesterase inhibitors or calcium channel blockers in our patients’ population. 

VS + patients had a trend towards longer invasive ventilation time compared to VS− patients (9 ± 11 vs. 5 ± 6 days; *p* = 0.06) (Table 3) and required more often renal replacement therapy (68% vs. 29.2%, respectively; *p =* 0.002) during the post-operative period. The intensive care unit length of stays was non significantly longer in VS+ compared to VS− patients (19.4 ± 24.2 vs. 13.3 ± 9.2 days; *p* = 0.12).

**Figure 1:**
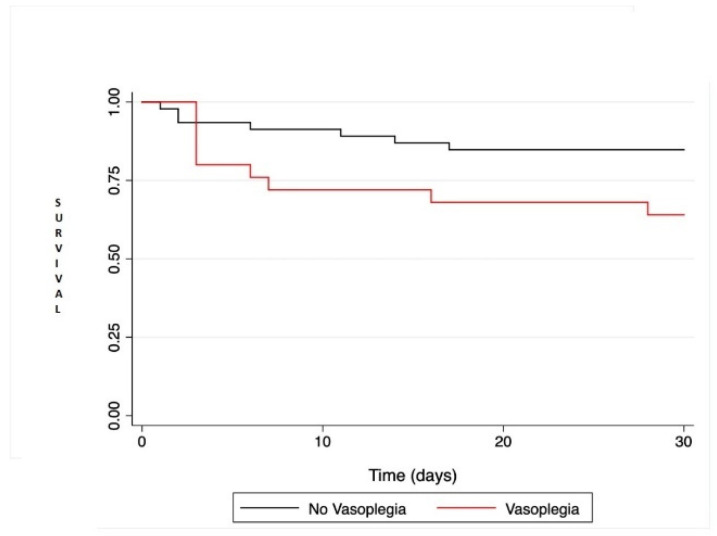
Kaplan-Meier survival analysis comparing patient survival at 30 days after heart transplantation or left ventricular assist device. Comparison between the two groups was performed with the log rank test and was not statistically significant (*p* = 0.051).

There was a trend towards increased mortality in VS+ patients compared to VS− (HR = 2.56; IC95% [0.95; 6.88]; *p* = 0.063) by univariate regression and Figure 1. All postoperative adverse events are reported in Table 3. 

### 3.4. Preoperative Vasoactive Treatment and 30-Day Outcomes

Thirty-day all-cause mortality was 13.6% (3/22 patients) in patients with pre-operative ARNI, 19.4% (6/31) in patients with other RASi, 33.4% (4/12) in patients on norepinephrine, and 62.5% (5/8) in patients with no vasoactive treatment (*p* = 0.043). 

**Figure 2:**
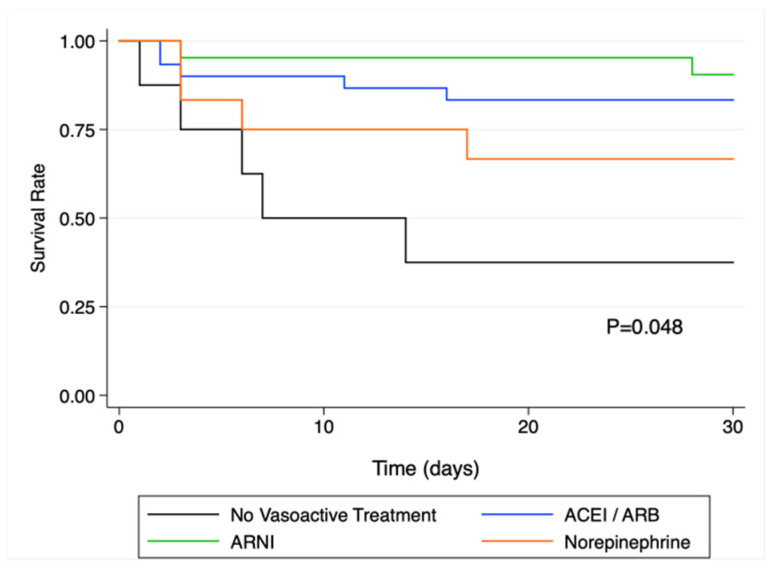
Kaplan-Meier survival analysis at 30 days in the different vasoactive treatment sub-groups. There was a significant difference in survival as assessed by the log rank test (*p* = 0.048), and survival was significantly altered in patients without vasoactive drugs prior to surgery. ACEi: angiotensin converting enzyme inhibitor; ARB: angiotensin receptor blocker; ARNi: angiotensin receptor-neprilysin inhibitors.

In the univariate Cox regression analysis, pre-operative treatment with ARNI, or other RAS inhibitors was associated with a significantly lower rate of death compared to patients with no vasoactive treatment (HR = 0.11; IC95% [0.02;0.55]; *p* = 0.009 for ARNI and HR = 0.20; IC95% [0.06; 0.69]; *p* = 0.011 for other RASi). The corresponding Kaplan and Meier curves are presented in Figure 2. 

In the multivariate regression analysis, only other RASi and on-pump duration time were independently associated with 30-day mortality. Cox multivariate regression analysis is presented in Table 4. 

## 4. Discussion

In this single tertiary center retrospective, observational study in HT and LVAD surgery patients, there was no significant association between pre-operative vasoactive medical treatment and the occurrence of vasoplegic syndrome. VS in this patient population had an incidence of 34% and was associated with increased rates of adverse events. Finally, pre-operative treatment with RAS inhibitors was independently associated with better survival at 30 days. 

Several observational studies have suggested that preoperative treatment with ACE inhibitors was associated with hypotension upon the induction of anesthesia [14,15,16,17]. As a result, the withdrawal of ACEI therapy before cardiac surgery has become a common practice [18]. Furthermore, current pharmacological management guidelines in cardiac surgery recommend stopping ARNI before the procedure. The angiotensin receptor–neprilysin inhibitors (ARNi) can counteract the effects of angiotensin II as well as to increase the activity of natriuretic peptides [19]. Compared with ACEI, angiotensin receptor-neprilysin inhibitor (ARNI), improved morbidity and mortality in patients with acute or chronic heart failure [8,20]. In these studies, ARNI was associated with significantly more hypotension compared to enalapril and valsartan alone [21].

The etiology of vasoplegia after cardiac surgery is not fully understood but likely multifactorial [2]. Generally, vasoplegia is a result of the activation of vasodilatory mechanisms and a resistance to vasopressors [2,5]. Patients who undergo cardiac transplantation may have additional factors that predispose them further to the development of vasoplegia. Advanced heart failure is associated with a proinflammatory state with an increased number of circulating inflammatory cytokines and cells. In addition, there is a more pronounced release of proinflammatory cytokines after cardiac transplant surgery as compared with conventional cardiac surgery [7].

Advanced heart failure is associated with a proinflammatory state with an increased number of circulating inflammatory cytokines and cells. In addition, there is a more pronounced release of proinflammatory cytokines after cardiac transplant surgery as compared with conventional cardiac surgery [2,3].

In our study, the incidence of VS in patients treated with ARNI was 40%, but this incidence was not significantly different from that of other drug groups. Our findings are similar to those reported in other studies. In the report by Dominguez et al. [22], preoperative ARNI treatment was not associated with an increased incidence of VS (7.2% vs. 17.1%) an overall 15.6% incidence of VS. The absence of significance in this setting might be related to the limited sample size and insufficient statistical power to demonstrate this association.

Although the guidelines of the United States and Europe recommend the administration of sacubitril/valsartan in NYHA class II-IV patients, analysis of real-world eligibility data suggests that only 20–40% of HFrEF patients will be eligible for sacubitril/valsartan initiation based on current guidelines [23]. ARNIs inhibit the RAS and enhance the activity of natriuretic peptides and bradykinin, thus demonstrating an effect on lowering blood pressure [24]. As predicted, hypotension was more prevalent in patients with sacubitril/valsartan than with enalapril in the PARADIGM-HF Trial [25].

Consistently with other series, vasoplegia is associated with delayed extubation, prolonged stay in the intensive care unit, multi-organ failure, and increased mortality [26,27,28]. Vasoplegia patients experienced longer intubation times; the combination of ongoing hemodynamic instability with vasopressor dependence and substantial fluid administration without adequate diuresis may have played a significant factor in preventing extubation from occurring at an earlier time point.

We observed an increased rate of adverse events and 30-day mortality in our patients with vasoplegia. Several studies have observed increased early morbidity and mortality in patients with vasoplegia following transplantation. Patarroyo observed a significantly higher incidence of re-exploration for bleeding and mediastinitis in patients with vasoplegia, and this was associated with a prolonged ICU and hospital length of stay and a significantly elevated 30-day mortality (17.1% vs. 3.2% *p* < 0.01) [28]. Similar findings were reported by Truby who observed a significantly elevated 30-day mortality in patients with vasoplegia (13.64% vs. 0.84% *p* < 0.01) [26].

Finally, to the best of our knowledge, this is the first study to suggest an improved 30-day survival rate with pre-operative RAS inhibition as compared to the absence of treatment. In secondary analyses, we found that the presence of blocker treatment of the renin-angiotensin system before a heart transplant or long-term cardiac support led to a decrease in the risk of 30-day mortality. This finding confirms the findings of other clinical trials [29,30] showing that RAS inhibitors reduce cardiovascular morbidity and/or mortality in patients undergoing a heart transplantation. Despite this, RAS blockade is still not recommended before cardiovascular surgery. Current randomized trials are underway to appropriately assess the pre-operative use of RAS blockade in patients undergoing heart surgery [31].

## 5. Limitations

The main limitation of this study is that it is retrospective and observational in nature and single center study with a relatively small number of patients, which limits the generalizability of our findings. The limited number of patients also impairs our ability to detect differences within subgroups. The presence of a non-reported confounder also increases the risk of bias.

All statistical tests that were performed here were exploratory, therefore our findings should be more considered as hypothesis-generating. Our data suggest, based on the higher prevalence of VS in the ARNI group with longer use of norepinephrine in this group, that there is a possible link between vasoplegia and ARNIs. However, there also is a significant improvement in adverse outcomes at 30 days in the ARNI group. The integration of those two results in contrast should encourage readers not to consider ARNI and VS in a simple bidimensional way. Our data generates new hypotheses on the impact of ARNI in patients undergoing cardiac surgery for transplant and LVAD. Further trials in larger groups of patients and in a prospective setting are needed to confirm these findings. Secondly, the lack of a consensus definition for vasoplegia limits direct comparison between our results and prior reports. Our definition of vasoplegia was aligned with that of similar published series, with a missing element to document the volemia status of patients which is another limitation of our report. However, all patients presenting with clinical evidence of hypovolemia in their medical files during the VS period were excluded from this analysis.

## 6. Conclusions

In this monocentric retrospective observational study, we did not observe a significant effect of sacubitril/valsartan on the incidence of VS post-transplant/LVAD surgery. In contrast, the use of sacubitril-valsartan in patients undergoing HT or LVAD was associated with improved 30-day survival. With the recent incorporation of sacubitril-valsartan into management guidelines for heart failure, and increasing clinical use, more studies with a larger number of patients are necessary to confirm our results.

## 7. Perspectives

COMPETENCY IN MEDICAL KNOWLEDGE 1: Vasoplegic syndrome after heart transplantation or left ventricular assist device surgery is associated with adverse outcomes.

COMPETENCY IN MEDICAL KNOWLEDGE 2: Pre-operative use of sacubitril valsartan before cardiac surgery has been associated with increased frequency in vasoplegic syndrome.

TRANSLATIONAL OUTLOOK 1: Preoperative use of sacubitril-valsartan was not associated with the development of vasoplegic syndrome in our patient population undergoing HT or LVAD surgery.

TRANSLATIONAL OUTLOOK 2: Our data suggests a significant 30-day survival benefit with efficient renin-angiotensin blockade prior to surgery in advanced heart failure patients undergoing heart transplant or LVAD surgery. Prospective randomized clinical trials in larger groups of patients are underway to validate these findings.

## Figures and Tables

**Table 1:** Baseline Characteristics of the Whole Study Population and patients with (VS+) and without (VS−) vasoplegia.

*Baseline Characteristics*	VS− (*n* = 48)	VS+ (*n* = 25)	Total (*n* = 73)	*p*
Age [years]	50.9 ± 11.5	53.3 ± 12.3	51.7 ± 11.7	0.41
Sex, male, n (%)	42 (87.5%)	23 (92%)	65 (89%)	0.71
BMI [kg/m^2^]	25.1 ± 4.1	25.9 ± 4.9	25.3 ± 4.4	0.47
LVEF < 35%, n (%)	45 (93.8%)	20 (80%)	65 (89%)	0.11
Euroscore II	15.8 ± 9.9	17.5 ± 12.7	16.4 ± 10.9	0.53
**Pre transplant treatment n (%)**				0.30
No vasoactive treatment	5 (10.4%)	3 (12%)	8 (11%)	
ACEI/ARB	24 (50%)	7 (28%)	31 (42.5%)	
ARNI	12 (25%)	10 (40%)	22 (30.1%)	
Norepinephrine	7 (14.6%)	5 (20%)	12 (16.4%)	
Betablockers	25 (52.1%)	13 (52%)	38 (52.1%)	1.00
**Initial Cardiac Disease, n (%)**				0.41
ICM	16 (33.3%)	12 (48%)	28 (38.4%)	
DCM	17 (35.4%)	6 (24%)	23 (31.5%)	
HCM	3 (6.3%)	2 (8%)	5 (6.8%)	
Two or more sternotomies	6 (12.5%)	6 (24%)	12 (16.4%)	0.32
**Type of surgery, n (%)**				
Transplantation	35 (72.9%)	19 (76%)	54 (74%)	1.00
LVAD	13 (27.1%)	6 (24%)	19 (26%)	1.00
Pump time, min	125.6 ± 45.5	125.6 ± 48.6	125.6 ± 40.1	0.91
GFR ml/min	60.9 ± 29.7	52.1 ± 26.0	57.9 ± 28.7	0.21
ECMO, n (%)	21 (43.8%)	13 (52%)	34 (46.6%)	0.62
Bleeding complications, n (%)	7 (14.6%)	7(28%	14 (19.2%)	0.21
**Septic event at 30 days, n (%)**				0.76
No sepsis	26 (54.1%)	12 (48%)	38 (52.1%)	
No documented sepsis	12 (25%)	6 (24%)	18 (24.7%)	
Documented sepsis	10 (20.83%)	7 (28%)	17 (23.3%)	

VS: vasoplegic syndrome, BMI: body mass index, LVAD: left ventricular assist device, GFR: glomerular filtration rate, calculated according to the CKD-EPI equation; ECMO: extracorporeal membrane oxygenation, RAS: Inhibitors of the renin-angiotensin system, ACEI: Angiotensin-converting enzyme inhibitors, ARBs: Angiotensin II receptor blockers; ARNi: angiotensin receptor-neprilysin inhibitor, LVEF: left ventricular ejection fraction. Comparison between VS+ and VS− patients was performed by parametric or non-parametric testing as appropriate.

**Table 2:** Post-operative use of norepinephrine.

	No Vasoactive Treatment (*n* = 8)	ACEI/ARBs (*n* = 31)	ARNI (*n* = 22)	Norepinephrine (*n* = 12)	*F*-Test
Norepinephrine duration, days	3.9 ± 2.3	4.7 ± 4.7	7.1 ± 6.8	10.3 ± 9.3	0.045
Norepinephrine, maximal dose mcg/kg/min	1.5 ± 1.4	1.0 ± 0.9	1.0 ± 0.6	1.1 ± 0.9	0.65

RASi: renin-angiotensin system inhibitors, ACEI: Angiotensin-converting enzyme inhibitors, ARBs: Angiotensin II receptor blockers, ARNI: angiotensin receptor and neprilysin inhibitors. All comparisons between groups are performed with an ANOVA, and the corresponding F-test result is presented.

**Table 3:** Postoperative adverse events according to the presence of VS.

Post-Operative Adverse Event	VS− (*n* = 48)	VS+ (*n* = 25)	*p*-Value
Death at 30-days n, (%)	9 (18.8)	9 (36.0)	0.051
Sepsis/infection n, (%)	22 (45.8)	13 (52.0)	0.61
Hemorrhagic complication n, (%)	7 (14.5)	7 (28)	0.17
Renal Replacement Therapy n, (%)	14 (29.2)	17 (68)	0.001
Mesenteric ischemia n, (%)	1 (2.08)	4 (16)	0.025
Peripheral ischemia n, (%)	0 (0)	2 (8)	0.047
Invasive ventilation time, days	5 ± 6	9 ± 11	0.06

**Table 4:** Multivariate regression analysis on factors associated with 30-day mortality.

	HR	95% CI	*p* Value
**Preoperative treatment:**			
- RASi	0.25	0.07–0.92	0.038
- ARNI	0.18	0.03–1.15	0.070
- Norepinephrine	0.42	0.11–1.65	0.217
Age	1.04	0.99–1.10	0.085
LVEF	0.76	0.18–3.16	0.707
ECS duration	1.02	1.01–1.04	0.010
Surgery type	2.53	0.51–12.56	0.255

HR: Hazar Ratio; RAS: Inhibitors of the renin-angiotensin system; ARNi: angiotensin receptor-neprilysin inhibitors; LVEF: Left ventricular ejection fraction; ECS: extra-corporeal support. For the pre-operative treatment group analysis, patients without any vasoactive treatment prior to surgery were considered as the reference group.

## Data Availability

Data presented in this study will be available for further analysis upon reasonable request to the lead investigators of this study and the Hospices Civils de Lyon.

## Supplementary Materials

The following are available online at https://www.mdpi.com/article/10.3390/medsci10010002/s1, Table S1: Baseline characteristics according to vasoactive patient category.
