# Supplementary material for: ARNI Pre-Operative Use and Vasoplegic Syndrome in Patients Undergoing Heart Transplantation or Left Ventricular Assist Device Surgery
Source: Med Sci (Basel). 2021 Dec 21;10(1):2. doi: 10.3390/medsci10010002 (PMC8788526; doi:10.3390/medsci10010002)
Supplement: Supplementary file 1 [file medsci-10-00002-s001.zip › medsci-1418474-supplementary.pdf]

Supplementary Material

Table S1: Baseline characteristics and outcomes in the different vasoactive groups of patients.

	No Vasoactive Treatment (n=8)	ACEI/ARBs (n=31)	ARNI (n=22)	Norepinephrine (n=12)
Age [years]	50.1±9.1	52.4±13.0	50.6±10.1	53.1±14.4
Sex, male	7(87.5%)	28(90%)	20(90.9%)	10(83.3%)
BMI [kg/m^2]	24.9±5.1	25.9±4.9	25.2±4.2	25.1±3.9
LVEF <35%	8(93.8%)	27(80%)	20(89%)	10(83.3%)
Euroscore II	18.7±9.2	15.5±10.7	16.1±10.1	16.8±9.9
Initial Cardiac Disease Diagnosis				
-ICM	3(37.5%)	12(38.7%)	9(40.9%)	4(33.3%)
-DCM	3(37.5%)	11(35.4%)	7(31.8%)	4(33.3%)
-HCM	1(12.5%)	2(6.5%)	2(9.1%)	1(8.3%)
-Other	1(12.5%)	6(19.4%)	4(18.2%)	3(25%)
Two or more sternotomies	1(12.5%)	5(16.1%)	4(18.2%)	2(16.7%)
Type of surgery				
-Transplantation	5(62.5%)	21(68.7%)	16(72.7%)	8(66.6%)
-LVAD	3(37.5%)	10(32.3%)	6(27.3%)	4(33.4%)
Pump time	130.6±40.5	122.1±45.3	125.1±46.2	126.1±38.5
GFR ml/min	48.0±13.2	54.1±17.6	62.1±21.0	55.6±18.7
ECMO	5(62.5%)	13(41.9%)	10(45.5%)	6(50%)
Bleeding complications	5(41.7%)	2(6.5%)	3(13.6%)	4(33.3%)
Documented sepsis within 30 days	3(37.5%)	5(16.1%)	5(22.7%)	4(33.4%)
Death at 30 days	5(62.5%)	6(19.4%)	3(13.6%)	4(33.4%)

Plus-minus values are means±SD. No statistical comparison was made between groups because of the prior multiple testing already performed in the manuscript, and the limited size of different sub-groups. significant differences be-tween the 2 groups for any variable. Percentages may not total 100 because of rounding. BMI indicates body mass index, LVEF left ventricular ejection fraction, ICM ischemic cardiomyopathy, DCM dilated cardiomyopathy, HCM hypertrophic cardiomyopathy, LVAD left ventricular assist device, GFR glomerular filtration rate, ECMO extra-corporeal membrane oxygenation.
